# Metagenomic diagnosis of severe psittacosis using multiple sequencing platforms

**DOI:** 10.1186/s12864-021-07725-9

**Published:** 2021-06-02

**Authors:** Kaiying Wang, Xiong Liu, Huiying Liu, Peihan Li, Yanfeng Lin, Dongdong Yin, Lang Yang, Jinhui Li, Shenlong Li, Leili Jia, Changqing Bai, Yongqiang Jiang, Peng Li, Hongbin Song

**Affiliations:** 1 Academy of Military Medical Sciences, Academy of Military Sciences, 20 Dongda Street, Fengtai District, 100071 Beijing, China; 2 Chinese PLA Center for Disease Control and Prevention, 20 Dongda Street, Fengtai District, 100071 Beijing, China; 3 Department of Respiratory and Critical Care Medicine, the Fifth Medical Center of PLA General Hospital, Beijing, China; 4 Anhui Province Key Laboratory of Veterinary Pathobiology and Disease Control College of Animal Science and Technology, Anhui Agricultural University, Hefei, China

**Keywords:** Metagenomic Sequencing, *Chlamydia psittaci*, Severe Pneumonia, Pathogen Identification

## Abstract

**Background:**

*Chlamydia psittaci* is an avian pathogen that can cause lethal human infections. Diagnosis of *C. psittaci* pneumonia is often delayed due to nonspecific clinical presentations and limited laboratory diagnostic techniques.

**Results:**

The MinION platform established the diagnosis in the shortest time, while BGISEQ-500 generated additional in-depth sequence data that included the rapid characterization of antibiotic susceptibility. Cytopathy appeared only in cell cultures of BALF. BALF yielded a higher bacterial load than sputum or blood, and may be the most suitable clinical specimen for the genomic diagnosis of severe pneumonia.

**Conclusions:**

This study indicated that the benefits of metagenomic sequencing include rapid etiologic diagnosis of unknown infections and the provision of additional relevant information regarding antibiotic susceptibility. The continued optimization and standardization of sampling and metagenomic analysis promise to enhance the clinical utility of genomic diagnosis.

**Supplementary Information:**

The online version contains supplementary material available at 10.1186/s12864-021-07725-9.

## Background

*Chlamydia psittaci* is a zoonotic intracellular pathogen that may cause fulminant disease in humans [1]. Genomic analysis has shown that *C. psittaci* is comprised of 15 genotypes [2, 3]. Genotype E isolates, first isolated from a human pneumonitis outbreak in the 1920 s, has been subsequently reported in a wide variety of hosts including pigeons, ratites, ducks, turkeys, and occasionally, humans [4, 5].

*C. psittaci* infection of humans, referred to as psittacosis, causes a spectrum of disease severity that encompasses asymptomatic transient carriage, mild pneumonia, and severe pneumonitis that can cause respiratory and multi-organ system failures, and in rare cases, death [6–9]. Clinical progression and mortality may be related to delays in diagnosis and treatment [4]. However, early diagnosis is confounded by nonspecific clinical presentations; low levels of clinical suspicion of an uncommon infection, and limited availability of diagnostic assays in most clinical laboratories [10, 11]. Cultures of *C. psittaci* are hazardous, time-consuming and require enhanced biosafety measures and expertise; serologic assays require acute and convalescent samples, and are thereby yield untimely results; and specific PCR has disadvantage that high sensitivity may depend on samples collected from the clinical phase [11].

Rapid diagnosis informs the prompt initiation of therapy to enhance clinical outcomes, and may also facilitate prompt source identification and contract tracing to prevent additional cases [12]. Metagenomic next-generation sequencing (mNGS) has been increasingly used for rapid and accurate diagnosis of infectious diseases including psittacosis [13, 14]. We report herein a case of fatal psittacosis diagnosed by mNGS. Our case illustrates that both the selection of specimen types and sequencing platforms are important in the diagnosis of severe pneumonia.

## Results

### Patient characteristics

**Fig. 1:**
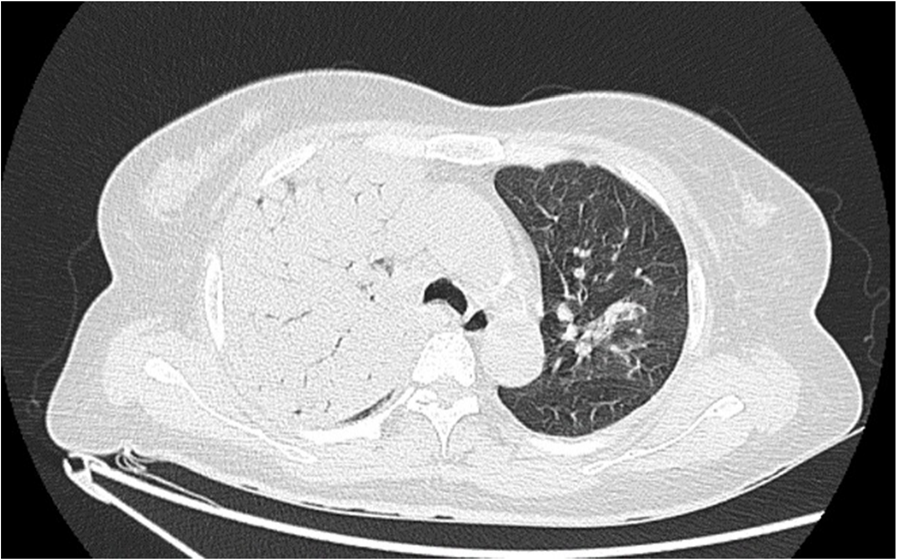
Chest computed tomography (CT) scan of a 60-year-old woman with severe pneumonia demonstrated large areas of consolidation in the right lung, ill-defined consolidation in the left lung, partial tracheobronchial stenosis in both lungs, and polyglandular mediastinal lymphadenopathy

A 60-year-old woman was admitted to the emergency department on July 25, 2019 with an eleven-day evolution of intermittent fever, non-productive cough, fatigue and arthralgia. Past medical history was notable for chronic hepatitis B infection, but was negative for nicotine, alcohol or drug abuse; unusual environmental or zoonotic exposures; or antecedent illnesses among family members. On the first hospital day, computerized tomography (CT) of the chest disclosed bilateral pulmonary infiltrates with dense consolidation of the right lung, polyglandular mediastinal lymphadenopathy, and partial tracheobronchial stenosis in both lungs (Fig. 1). Additional findings included a leukocyte count of 15.12 × 10^9^/L (reference range: 3.5–9.5 × 10^9^/L) with 95.9 % neutrophils (40–75 %), platelet count 47 × 10^9^/L (100–300 × 10^9^/L); and C-reactive protein 564 mg/L (0-8 mg/L). Antibiotic therapy was initiated with moxifloxacin, tigecycline, and imipenem. On the second hospital day, the patient underwent endotracheal intubation and mechanical ventilation indicated for respiratory failure. The antibiotic regimen was revised to moxifloxacin plus tigecycline after *C. psittaci* was identified on the second hospital day. However, the patient’s condition continued to deteriorate, and she died on the fourth hospital day.

### Pathogen identification, isolation and verification

From the sample receipt to the results, it took 11 h for the MinION platform, 24 h for the Illumina platform, and approximate 200 h for the BGI platform. The read length of BGI and Illumina platforms is 100 and 75 bp, respectively, while MinION generated reads with lengths ranged from 35 bp to 26,140 bp. Specific reads obtained from all three sequencing platforms identified *C. psittaci* as the dominant pathogen. Cytopathy was observed only in the BALF culture (Fig. S1). PCR assays identified *C. psittaci* only in the BALF culture, whereas cell blood and sputum cultures were PCR-negative (Table S1). We designated our isolate *C. psittaci* strain L99; genomic analysis indicated that it belonged to *ompA* genotype E (Fig. S2).

### Phylogenetic analysis of ***Chlamydia psittaci*** strain L99

**Fig. 2:**
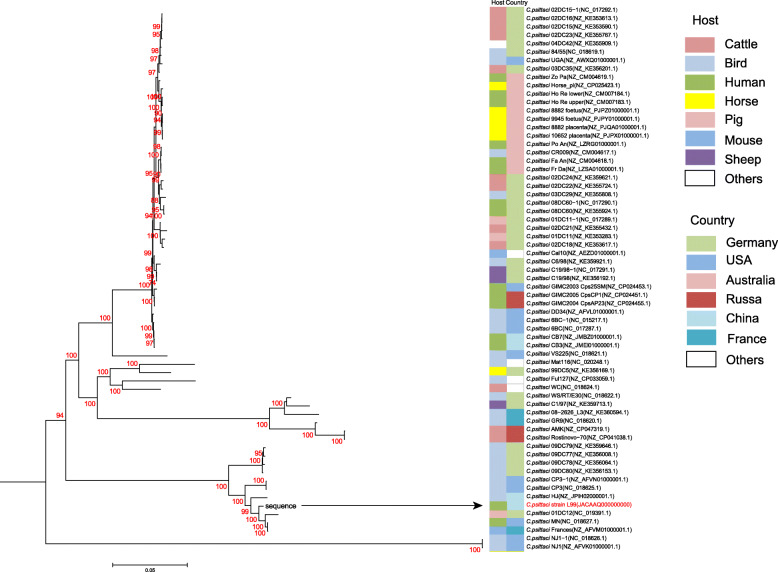
Whole-genome phylogeny of *C. psittaci* strain L99. The phylogenetic tree was constructed by the maximum likelihood method with bootstrap analysis (*n* = 1000) The left side represents the host of *C. psittaci* and the right side represents the country of *C. psittaci*, which are distinguished by different colors. The *C. psittaci* strain L99 is marked with a black arrow and red font. The values of internal nodes with bootstrap support greater than 70 % are shown above branches

Genomic analysis of *C. psittaci* strain L99 was conducted on the six data sets. No single nucleotide polymorphisms were identified among the three platforms. the draft genome of *C. psittaci* was assembled into 34 contigs using all of the sequencing data. The patient’s exposure history provided no information to implicate the source of *C. psittaci*. Phylogenetic analysis suggested that *C. psittaci* strain L99 was related to *C. psittaci* MN(NC_018627.1), a human isolate from the USA [15]; and *C. psittaci* Strain 01DC12 (NC_019391.1), from swine in the Germany [16] (Fig. 2). However, MLST analysis showed that *C. psittaci* strain L99 belonged to sequence type ST35, which is consistent with *C. psittaci* MN rather than Strain 01DC12 (ST56). In all sequencing data, no resistance gene and virulence factor were identified in *C. psittaci* L99 genome.

### Divergent sequencing data between platforms and samples

Only host- and *C. psittaci*-specific sequences were recognized by MinION, however, species identified by the BGISEQ-500 and Illumina platforms were quite diverse. Species detected in the same sample types were quite different between these two platforms (Table S2). Besides of dominant *C*. *psittaci*, both platforms identified *Propionibacterium acnes* and *Klebsiella pneumoniae* in sputum and blood samples. However, there were no significant inter-platform differences in pathogens identified in the different sample types.

*C. psittaci* strain L99 was the only pathogen identified in all six data sets. The BGISEQ-500 platform generated more data and provided the most comprehensive genomic information on unique read numbers, coverage, and sequencing depth of *C. psittaci* strain L99 than either the MinION or Illumina platforms (Fig. S3). The average read depth of *C. psittaci* strain L99 obtained for different samples from each platform was displayed in Fig. S4. The BGI platform generated an average read depth of 6.84×, 5.25× and 2.79× for the BALF, sputum and blood samples, respectively. Illumina generated an average depth of 1.50× and 1.21× for the sputum and blood sample, while MinION had an average depth of 1.00× for the BALF sample.

### Divergent results on the BGISEQ-500 platform

**Fig. 3:**
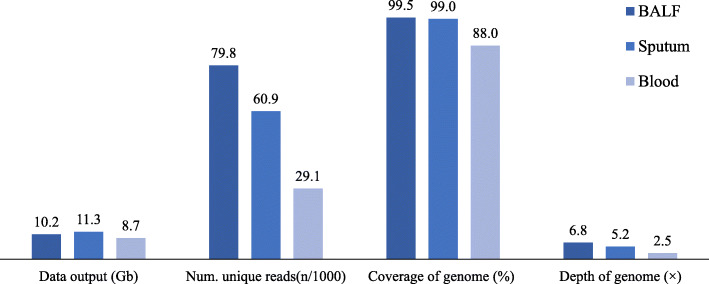
Difference of sequencing data of *Chlamydia psittaci* strain L99 in multiple samples on BGISEQ-500 platform. The colors from dark to light represent the three data sets of BALF, sputum, and blood, respectively. The four sets of data include data output of sample, number of unique reads (n/1000) of *C. psittaci* strain L99, coverage of *C. psittaci* strain L99 genome, and depth of *C. psittaci* strain L99 genome

The BGISEQ-500 platform yielded results that differed according to sample type. Coverages of the *C. psittaci* genome in BALF, sputum and blood were 99.5 %, 99 and 88 %, respectively. The depth of the *C. psittaci* genome decreased in BALF, sputum, and blood, respectively (Fig. 3). Of the top ten identified species, eight pathogens were detected in all three sample types (Table 1). *C. psittaci* strain L99 was the most abundant pathogen in all sample types.

**Table 1:** Top ten species in three sample types by BGISEQ-500

Top ten species	Number of unique reads(n)		
	BALF	Sputum	Blood
*Chlamydia psittaci*^a^	39,385	30,320	14,478
*Propionibacterium acnes*^a^	64	59	171
*Stenotrophomonas maltophilia*^a^	54	102	150
*Chlamydia abortus*	46	23	/
*Klebsiella pneumoniae*^a^	29	22	24
*Salmonella enterica*^a^	27	56	43
*Pseudomonas aeruginosa*^a^	20	16	29
*Moraxella osloensis*^a^	17	9	26
*Micrococcus luteus*	10	8	/
*Escherichia coli*^a^	8	10	50
*Enterococcus faecium*	/	/	11
*Staphylococcus epidermidis*	/	/	11

^a^indicates species that were detected in all three sample types. “/” indicates species that were not among the top ten species of the corresponding sample

## Discussion

We identified *C. psittaci* by applying mNGS to multiple sample types from a patient with severe pneumonia. BALF was the only sample that yielded *C. psittaci* by traditional cell culture, while mNGS results of all three samples were diagnostic. Our study demonstrated mNGS has the advantages of rapidity and high sensitivity when applied to unknown infections. And compared with specific PCR, mNGS could also provide information about resistance genes or genetic markers that may facilitate clinical treatment and epidemiologic investigations [17, 18].

Phylogenetic analysis disclosed that *C. psittaci* strain L99 was closely related to *C. psittaci* MN(NC_018627.1), isolated from an American case of psittacosis. However, our patient’s medical history was negative for travel, zoonotic, or other significant exposures. Our case shows that the epidemiologic sources of psittacosis cases may not always be evident. Consequently, it is necessary to educate the populace, healthcare providers, and public health officials on psittacosis to facilitate early diagnosis and epidemiologic investigations.

In the identification of multiple bacterial species, there are some sequences of *C. abortus* and *C. felis*, which belong to the same genus as *C. psittaci*. Analysis on the reads matching *C. abortus* or *C. felis* revealed that these reads can be aligned to *C. psittaci* but with higher identity. The species classification of metagenomics sequencing reads by Centrifuge should be carefully reviewed.

We found that BALF was the most sensitive of the three specimen types for isolation and detection. Metagenomic diagnosis in clinical infection showed that genome coverage of *C. psittaci* in BALF (0.5012 %) was about 2.86-fold higher than in blood (0.1755 %) [14]. In our study, the genome coverage of *C. psittaci* in BALF was as high as 99.5 %. Therefore, BALF may be considered the specimen of first choice for the diagnosis of *C. psittaci* pneumonia.

## Conclusions

In this study, through the comparison of different platforms and analytes, MinION provides real-time sequencing and long reads but with limited output and high cost, while the BGI platform can generate sufficient data and provide more information with lower cost but longer time. The Illumina platform had a performance between MinION and BGI on time and cost, which is prevalent in clinical applications. Compared with serology and multiplex PCR which can only detect known pathogens, the continuous optimization and cost reductions of mNGS, combined with appropriate sample selection, can promote rapid diagnosis and provide more clinically broad-spectrum pathogens and epidemiologically relevant information, especially in unknown infections.

## Methods

### Sample collection and nucleic acid extraction

Lower respiratory tract specimens (bronchoalveolar lavage fluid [BALF] and sputum) and blood were collected for nucleic acid extraction. For MinION and BGISEQ-500 library preparation, BALF and blood were centrifuged at 3000 rpm for 20 min under 4℃ to collect supernatant. Sputum was digested by 1 % trypsin at 37℃ for 30 min. 500µL supernatants and 500µL digested sputum were used for nucleic acid extraction using the QIAamp MinElute Virus Spin Kit (Cat No: 57,704, Qiagen, USA) according to the manufacturer’s instructions, respectively. RNA was transformed into double-stranded DNA with NEBNext Ultra II RNA First Strand Synthesis Module (Cat No: E7771, NEB) and NEBNext Ultra II Non-Directional RNA Second Strand Synthesis Module (Cat. No: E6111, NEB) according to the manufacturer’s instructions.

Aliquots of blood and sputum were sent to Vision Medicals (sequencing company, Beijing) for sequencing. Nucleic acids were extracted using TIANamp Micro DNA Kit (DP316, TIANGEN BIOTECH, China) following the manufacturer’s operational manual.

### Metagenomic sequencing and analysis

Library preparation and sequencing kits were selected according to corresponding sequencing platforms. Kit details are shown in Table S3. The entire processes were carried out according to the manufacturer’s protocols. The DNA preparing for library were sheared into 200 bp with micro-TUBE (Cat. No: 520,045, Covaris, USA) on Covaris M220 for Illumina platform and 300 bp by fragmentase for BGI platform. There was no fragmentation during library preparation for MinION platform.

Quality control and removal of low-quality reads were performed by PycoQC v2.2.4 [19] and SOAPnuke v2.0.7 [20], respectively. Clean reads were classified by Centrifuge v1.0.4 [21]. Bowtie 2 (version 2.3.5.1) [22] and MEGAHIT (version 1.2.9) [23] were used for read alignment and genome assembly, respectively.

The maximum likelihood phylogenetic tree of *C. psittaci* strain L99 and other 22 previously described global *C. psittaci* strains from NCBI was constructed by kSNP3.1 [24] with bootstrap analysis (n = 1000). Seven genes of strain L99 (*enoA*-*fumC*-*gatA*-*gidA*-*hemN*-*hflX*-*oppA*) were used for genotyping through the *Chlamydiales* MLST database [25]. Resistance genes and virulence factors were identified by BLAST analysis of the assembled sequences against the Comprehensive Antibiotic Resistance Database and the Virulence Factors Database with cutoffs of 95, respectively.

### Pathogen verification

Vero cell cultures were inoculated directly with BALF, sputum, and blood samples, and incubated for 4 days. Cytopathy was observed by light microscope. Vero cells were collected for nucleic acid extraction using the QIAamp MinElute Virus Spin Kit (Cat No: 57704, Qiagen) according to the manufacturer’s manual. DNA was processed to detect the *C. psittaci* outer membrane protein A (*ompA*) gene by PCR using primers ompA-F (5’-ACTATGTGGGAAGGTGCT-3’) and ompA-R (5’-TAGACTTCATTTTGTTGATCTGA-3’) [26]. The products were used for agarose gel electrophoresis and Sanger sequencing. Nuclease-free water was used as a negative control. The sequence of PCR product was evaluated by BLAST analysis against the NCBI database and used for typing by DNAStar with representative genotype sequences of *C. psittaci*.

## Supplementary Information

**Additional file 1.**

## Data Availability

The whole-genome sequence of *C. psittaci* strain L99 has been deposited in GenBank under accession number JACAAQ000000000.
