# Supplementary material for: Metagenomic diagnosis of severe psittacosis using multiple sequencing platforms
Source: BMC Genomics. 2021 Jun 2;22:406. doi: 10.1186/s12864-021-07725-9 (PMC8173916; doi:10.1186/s12864-021-07725-9)

**Supplementary Materials**

**Table** S1 Detection of *C. psittaci* in different methods and samples

| Sample | MinIon | Illumina | BGI | Cell culture | PCR |
| --- | --- | --- | --- | --- | --- |
| BALF | Positive | - | Positive | Positive | Positive |
| Blood | - | Positive | Positive | Negative | Negative |
| Sputum | - | Positive | Positive | Negative | Negative |

“-” indicates the sample was detected without that method.

**Table S2** Top ten species in sputum and blood samples of BGISEQ-500 and Illumina

| Number of unique reads(n) of species in sputum | | | |
| --- | --- | --- | --- |
| Top ten species | BGI | Top ten species | Illumina |
| *Chlamydia psittaci* | 30320 | *Chlamydia psittaci* | 8555 |
| *Stenotrophomonas maltophilia* | 102 | *Propionibacterium acnes* | 44 |
| *Propionibacterium acnes* | 59 | *Sphingomonas melonis* | 43 |
| *Salmonella enterica* | 56 | *Chlamydia abortus* | 13 |
| *Chlamydia abortus* | 23 | *Mycoplasma wenyonii* | 10 |
| *Klebsiella pneumoniae* | 22 | *Pseudomonas sp. JY-Q* | 8 |
| *Pseudomonas aeruginosa* | 16 | *Staphylococcus epidermidis* | 5 |
| *Escherichia coli* | 10 | *Delftia tsuruhatensis* | 4 |
| *Moraxella osloensis* | 9 | *Acinetobacter johnsonii* | 4 |
| *Micrococcus luteus* | 8 | *Klebsiella pneumoniae* | 3 |
| Number of unique reads(n) of species in blood | | | |
| Top ten species | BGI | Top ten species | Illumina |
| *Chlamydia psittaci* | 14478 | *Chlamydia psittaci* | 2227 |
| *Propionibacterium acnes* | 171 | *Sphingomonas melonis* | 13 |
| *Stenotrophomonas maltophilia* | 150 | *Mycoplasma wenyonii* | 7 |
| *Escherichia coli* | 50 | *Chlamydia felis* | 3 |
| *Salmonella enterica* | 43 | *Propionibacterium acnes* | 3 |
| *Pseudomonas aeruginosa* | 29 | *Human herpesvirus 4 type 2* | 3 |
| *Moraxella osloensis* | 26 | *Chlamydia abortus* | 2 |
| *Klebsiella pneumoniae* | 24 | *Acinetobacter baumannii* | 2 |
| *Staphylococcus epidermidis* | 11 | *Klebsiella pneumoniae* | 2 |
| *Enterococcus faecium* | 11 | *Ralstonia pickettii* | 2 |

**Table S3** Kits and devices of library preparation and sequencing

| Platform | Kit of library preparation and metagenomic sequencing | |
| --- | --- | --- |
| MinION | Rapid Sequencing Kit (Cat. No: SQK-RAD004, Oxford Nanopore Technologies) | Flow Cell (FLO-MIN106 R9 Version) |
| BGISEQ-500 | MGIEasy DNA Rapid Library Prep Kit (Cat No: 940-200033-00, MGI, China) | BGISEQ-500RS High-throughput Sequencing Set PE100 V3.0 |
| Illumina NextSeq 500 | VAHTS^TM^ Universal DNA Library Prep Kit (Cat. No: ND606, Vazyme, China) | NextSeq 500/550 Mid Output Kit v2 |


**Figure legends**

**Figure S1** Cytopathology of Vero cells inoculated with BALF. A is the control (Vero cells) and B is the Vero cells inoculated with BALF.

**Figure S2** Gene *ompA* of *Chlamydia psittaci* phylogenetic analysis. The neighbor-joining tree was constructed by DNAStar. The *ompA* gene of *C. psittaci strain L99* is labeled with red font.

**Figure S3** Analysis of *Chlamydia psittaci* between different sequencing platforms. From left to right: data output of the sample (Gb), number of unique reads (n/1000), coverage of genome (%) and depth of genome (×), respectively. **A** is the difference of *C. psittaci* from BALF between MinION (dark blue) and BGISEQ-500 (light blue) platforms. **B** is the difference of *C. psittaci* from sputum between BGISEQ-500 (dark green) and Illumina (light green) platforms. **C** is the difference of *C. psittaci* from blood between BGISEQ-500(dark gray) and Illumina (light gray) platforms.

**Figure S4** Average reads depth of *Chlamydia psittaci* between different sequencing platforms and samples. X axis is base position of *C. psittaci* 01DC12 genome and Y axis is the reads depth. The average read depth of different samples and platforms was distinguished with different color lines. The value of average read coverage depth from high to low is 6.84× of the BGI_BALF, 5.25× of BGI_ sputum, 2.79× of BGI_blood, 1.50× of Illumina_sputum, 1.21× of Illumina_blood and 1.00× of MinION_BALF, respectively.

**Figure S1**


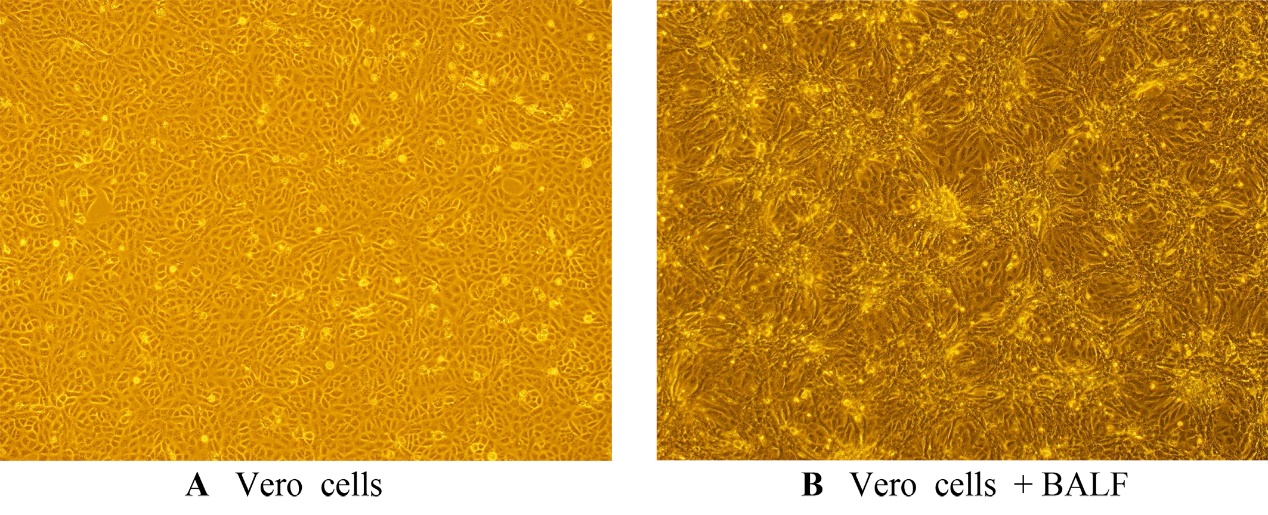


**Figure S2**


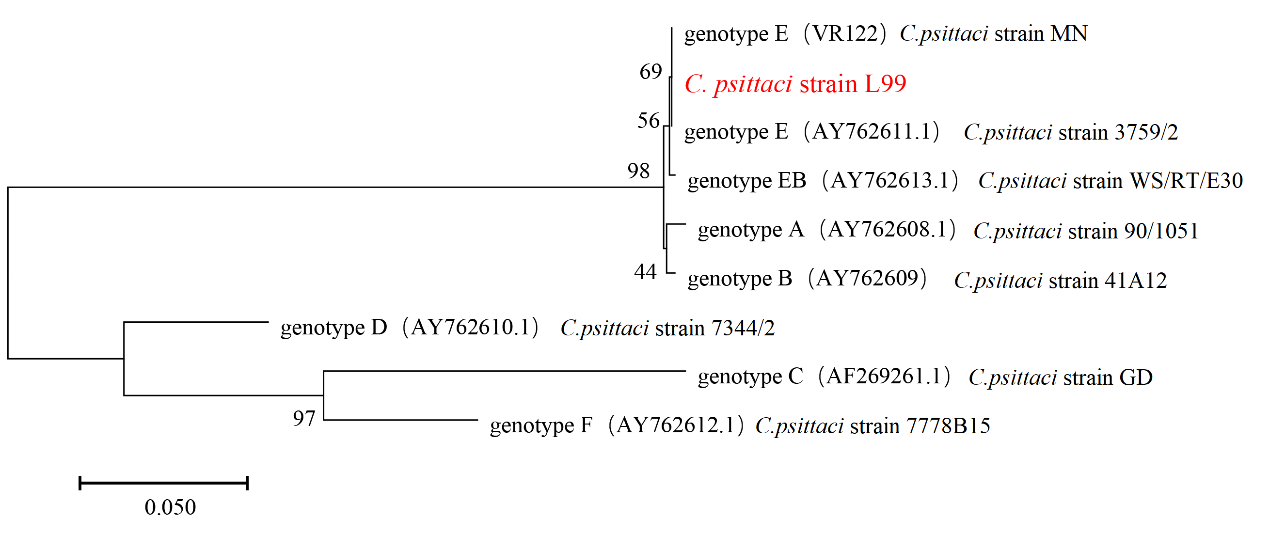


**Figure S3**


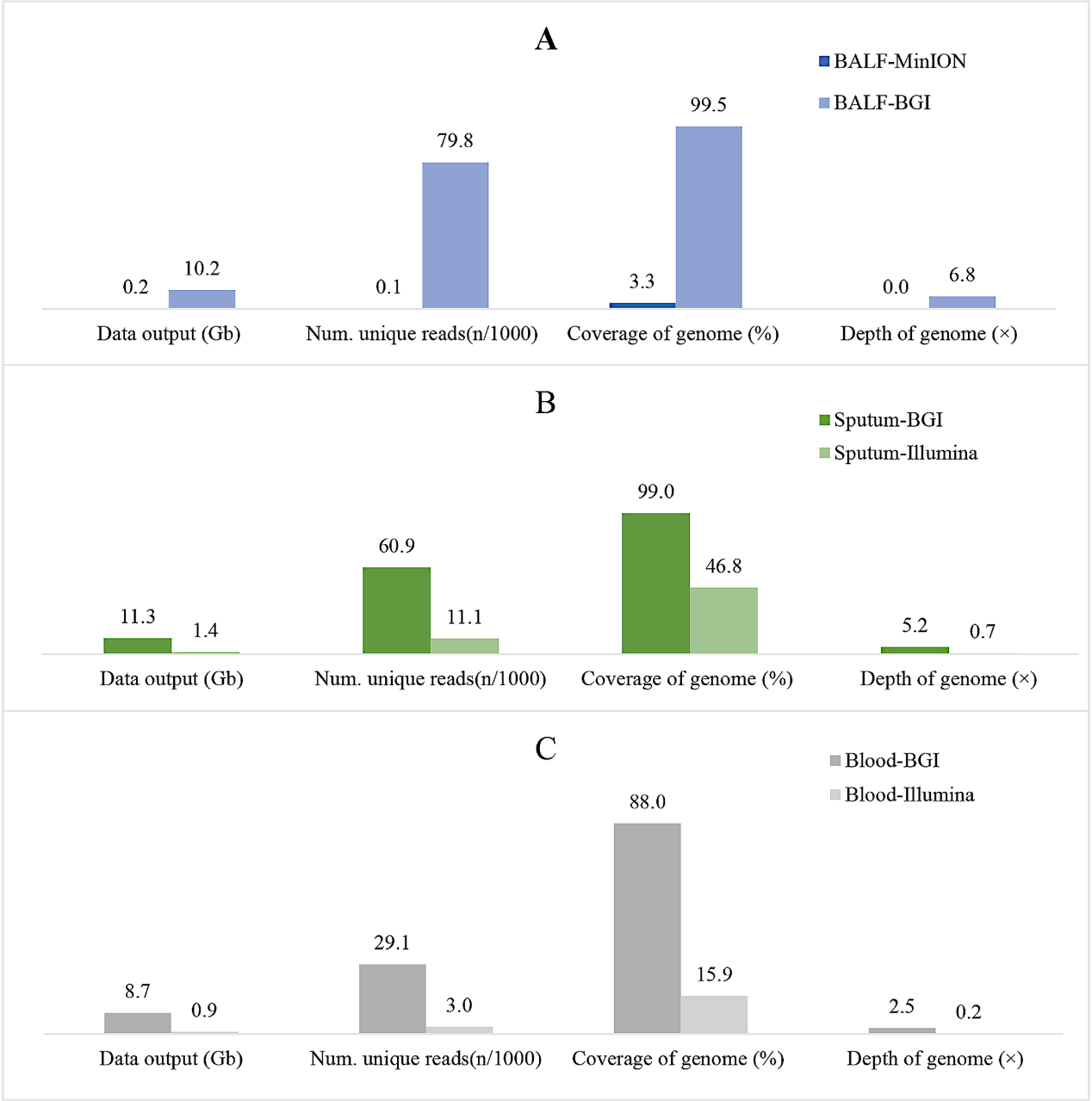


**Figure S4**


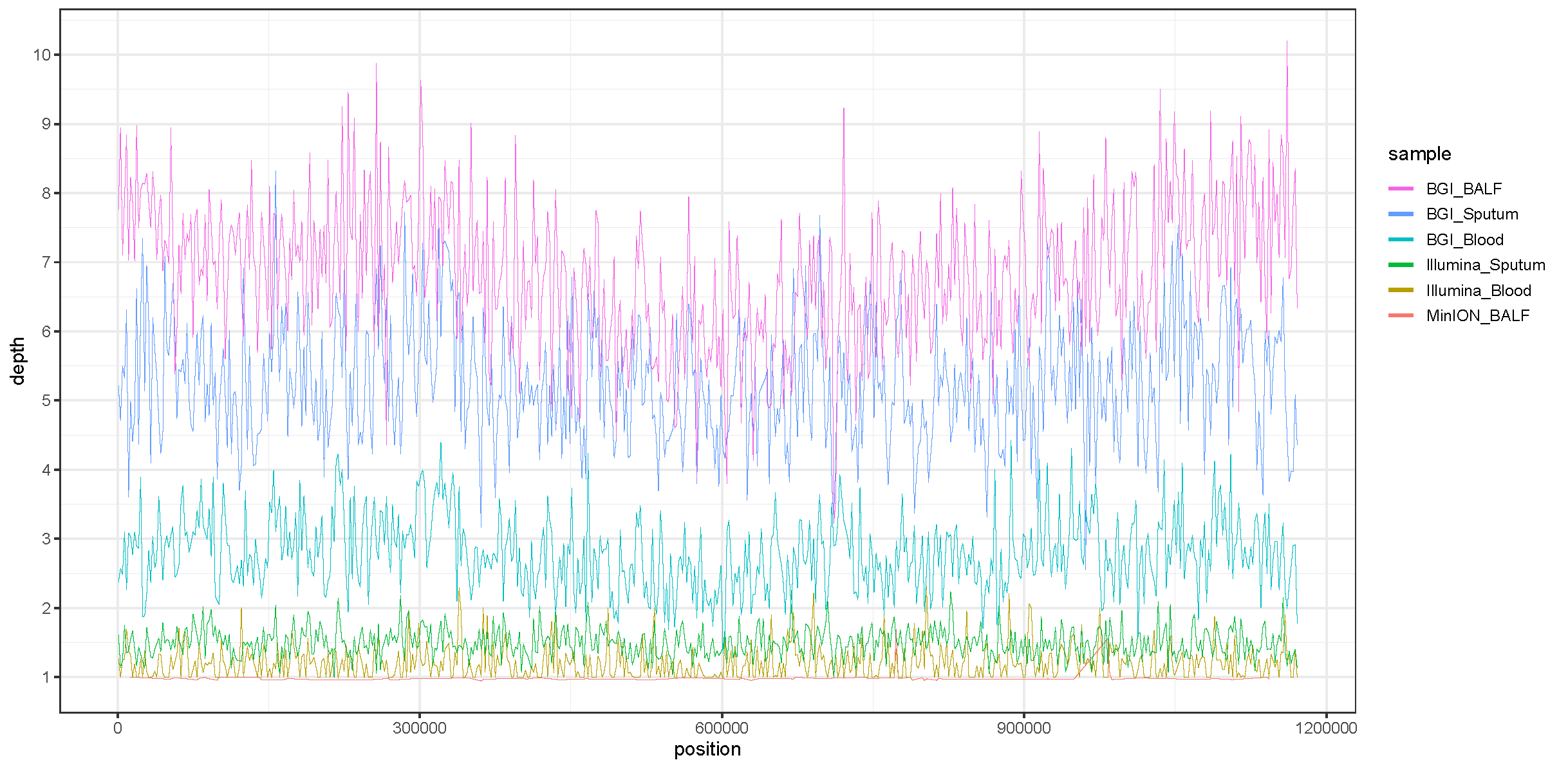

Supplement: Supplementary file 1 — Additional file 1. [file 12864_2021_7725_MOESM1_ESM.docx]
